# The molecular pathways underlying host resistance and tolerance to pathogens

**DOI:** 10.3389/fgene.2012.00263

**Published:** 2012-12-14

**Authors:** Elizabeth J. Glass

**Affiliations:** The Roslin Institute and Royal (Dick) School of Veterinary Studies, University of EdinburghEdinburgh, UK

**Keywords:** genetics, breeding, disease resistance, tolerance, livestock, immunity, inflammation, pathogen

## Abstract

Breeding livestock that are better able to withstand the onslaught of endemic- and exotic pathogens is high on the wish list of breeders and farmers world-wide. However, the defense systems in both pathogens and their hosts are complex and the degree of genetic variation in resistance and tolerance will depend on the trade-offs that they impose on host fitness as well as their life-histories. The genes and pathways underpinning resistance and tolerance traits may be distinct or intertwined as the outcome of any infection is a result of a balance between collateral damage of host tissues and control of the invading pathogen. Genes and molecular pathways associated with resistance are mainly expressed in the mucosal tract and the innate immune system and control the very early events following pathogen invasion. Resistance genes encode receptors involved in uptake of pathogens, as well as pattern recognition receptors (PRR) such as the toll-like receptor family as well as molecules involved in strong and rapid inflammatory responses which lead to rapid pathogen clearance, yet do not lead to immunopathology. In contrast tolerance genes and pathways play a role in reducing immunopathology or enhancing the host's ability to protect against pathogen associated toxins. Candidate tolerance genes may include cytosolic PRRs and unidentified sensors of pathogen growth, perturbation of host metabolism and intrinsic danger or damage associated molecules. In addition, genes controlling regulatory pathways, tissue repair and resolution are also tolerance candidates. The identities of distinct genetic loci for resistance and tolerance to infectious pathogens in livestock species remain to be determined. A better understanding of the mechanisms involved and phenotypes associated with resistance and tolerance should ultimately help to improve livestock health and welfare.

## Introduction

Selective breeding strategies for livestock species have been employed to great advantage for the human race, creating new breeds with improved productivity traits such as increased milk yield and faster growth. This process has gained momentum in recent decades with advances in technologies and resources to achieve more targeted breeding. Thus, the process of selection in species of agricultural importance has changed from relying on readily observable phenotypes e.g., coat color, to employing high density SNP chips and genomic prediction of specific production traits (reviewed by Hume et al., 2011). We are now experiencing a genomics information explosion with the advent of cheaper and faster sequencing of genomes. Aims currently include sequencing multiple individuals within species including a project to sequence 1000 cattle genomes (www.1000bullgenomes.com). In theory, this type of information could provide sufficient knowledge and resources for genetic variation that could ever be needed to target selective breeding for specific traits. Furthermore, strong evidence has accumulated that livestock species from birds to mammals, harbor genes that control protective responses to the various classes of pathogen from viruses to complex metazoans such as nematodes (for reviews that comprehensively cover different livestock species and pathogens see e.g., Davies et al., 2009; Mirkena et al., 2010). However, there remains a “phenotype gap” for traits linked to disease resistance and tolerance (Glass et al., 2012a,b). This is partly because host-pathogen interactions are highly complex, involving many different molecules and cell types which interact together over time. Invoking the wide arsenal of defense mechanisms in the host is partly dependent on the pathogen and partly on other factors such as the physiological state of the animal as well as previous exposure. Thus, the outcome—protection and survival or disease and potentially death—and the relationship of these to a measurable outcome of fitness, which in the case of livestock is usually equated to traits such as growth or yield, is difficult to predict. Furthermore, the correlates of protection or pathogenesis are often unclear and even under more straightforward situations, the logistical difficulties of measuring relevant phenotypes at the most appropriate time points in livestock in the field can be formidable. In addition, many factors, both genetic and non-genetic influence the outcome of exposure to pathogens. Nowhere is this more of an issue than considerations of what parameters to measure in order to ascertain whether an animal is resistant or tolerant to a pathogen. This article will explore from an immunologist's point of view, the definitions of “resistance” and “tolerance,” how to measure them, and whether they are separately determined traits controlled by many non-overlapping genes. The main focus is on identifying the genes and molecular pathways that underpin host defense from pathogens, and are likely candidates for resistance and tolerance traits.

## Resistance and tolerance: definition and fitness trade-offs

In the context of this review and special issue, resistance is defined as an ability to reduce pathogen replication in the host, whereas tolerance is defined as an ability to maintain homeostasis in the presence of a replicating pathogen, with limited ensuing pathology (see Doeschl-Wilson et al., 2012a,b). Thus, resistance traits may be considered as those governed by genes that function as barriers to pathogen entry as well as genes expressed during an active innate or acquired immune response to the pathogen that result in a reduction of pathogen burden. In contrast, tolerance traits may be controlled by genes that suppress or otherwise limit active responses to the pathogen and/or genes that prevent pathogen mediated toxicity, but have no effect on pathogen burden. Indeed, many diseases are caused by collateral damage of the host's own tissues during the process of immune-related defense mechanisms, i.e., immunopathology, rather than toxicity caused by the pathogen itself. For example, young cattle infected with bovine respiratory syncytial virus (BRSV), show considerable lung pathology that appears to be linked to an influx of immune cells, whereas infection of bovine epithelial cells *in vitro* with BRSV do not show cytopathology (Valarcher and Taylor, 2007). Although there is considerable literature to suggest that, there is a genetic component to the response to BRSV (Glass et al., 2010, 2012a; Leach et al., 2012), whether this relates to resistance and/or tolerance is unclear. Additionally, an ability to protect the host from damage caused by pathogen derived toxicity could also be a component of a tolerance phenotype (Medzhitov, 2009).

Thus, for a host species to survive there needs to be a balance between protection against the onslaught of infection, and the consequences of immunopathology and direct toxicity by the pathogen. The selective pressure exerted by pathogens on their hosts drives the evolution of counter-measures and vice versa (Woolhouse et al., 2002). This co-evolution may result in the development of resistance or tolerance mechanisms in the host (Carval and Ferriere, 2010), and virulence factors (Ebert and Bull, 2003) or ways of evading or subverting the host immune defense mechanisms in the pathogen (Schmid-Hempel, 2009). These host-pathogen interactions across time leave their mark on the host genome in terms of polymorphisms in genes underpinning resistance and tolerance traits. However, the complexity of these interactions together with heterogeneous environmental factors makes it difficult to predict optima or outcomes (Lazzaro and Little, 2009).

The evidence that different genes control disease resistance and tolerance was originally obtained from plant studies, which demonstrated that genetic variation in both resistance and tolerance existed (Simms and Triplett, 1995). Gene variants that confer greater resistance to pathogens are predicted to be unlikely to go to fixation in a population, because although they effectively reduce the levels of pathogen burden, their fitness costs outweigh the costs of retaining the resistance traits in the absence of infection (Roy and Kirchner, 2000). In contrast, if a host evolves more effective tolerance mechanisms, it has been hypothesized that these would no longer act as further selective pressure on the pathogen (Roy and Kirchner, 2000). Increased frequencies of tolerant individuals would lead to a rise in pathogen burden in a population, and thus, any fitness benefits of tolerance are predicted to drive tolerance traits to fixation (Roy and Kirchner, 2000). The eventual outcome in such cases might achieve an equilibrium in which host populations become completely tolerant to the surrounding pathogens. These may then be observed as endemic or even as commensals or environmental micro-organisms (Medzhitov, 2009; Nussbaum and Locksley, 2012).

However, these conclusions make assumptions that resistance always confers a negative fitness cost and that tolerance always confers a positive cost, whereas in plant studies, the estimated costs of resistance and tolerance do not necessarily follow evolutionary theory in that fitness is not necessarily compromised by resistance to pathogens, nor is tolerance necessarily beneficial to fitness (Simms and Triplett, 1995). Thus, it has been argued that the relationship between resistance and tolerance is dependent on the trade-offs each impose on the host in terms of fitness (Restif and Koella, 2004; Carval and Ferriere, 2010).

The trade-offs also depend on the virulence of the infecting organism, which raises another consideration: virulence of the infecting organism is intimately associated with the response by its host (Margolis and Levin, 2008). The definition of virulence is still widely debated in the literature, with the debate ranging from the micro-organism perspective to the host. Many define virulence as the ability of a micro-organism to multiply in a host and cause harm (Poulin and Combes, 1999) i.e., the capacity to infect and ability to transmit, which relates to pathogen fitness (Kirchner and Roy, 2002). However, virulence in relation to animals is commonly defined as a pathogen-induced reduction in host fitness, which is dependent on pathogen dose and is therefore, a consequence of host-pathogen interactions (Casadevall and Pirofski, 2001; Margolis and Levin, 2008). This is the definition used in this review. Virulence is usually measured by the level of host mortality, but often for practical and ethical reasons the degree of host morbidity is used instead (Alizon et al., 2009). However, virulence can be attributed to both intrinsic virulence factors of the micro-organism, which can cause direct toxicity as well as damage caused by the host response to the micro-organism (Day et al., 2007; Best et al., 2012). Furthermore, what is harmful to one host species may exist as a commensal in another species (Casadevall and Pirofski, 2001). Thus, for example, *Escherichia coli 0157* or *Salmonella spp* are carried and shed by livestock with little apparent ill effect on these host species (Stevens et al., 2009; Clermont et al., 2011). However, these micro-organisms can cause serious consequences in humans and indeed in young or old livestock. This highlights the fact that the *same* micro-organism can have very different effects-dependent on the host species and its physiological state. Further the consequences of infection are also determined by the micro-organism's route of entry or translocation from one host niche to another. For example, many commensal gut bacteria only cause disease when the gut epithelia is compromised (Pamer, 2007) or the human nasopharynx commensal, *Neisseria meningitides*, only causes severe meningitis or septicaemia if it is able to invade other tissue compartments (Trivedi et al., 2011).

Moreover, another reason that resistance or tolerance genes might not go to fixation in populations may be because the gene variants predispose carriers to infection with a different pathogen (Dean et al., 2002). Indeed, Ayres and Schneider (2008) found that when they infected a mutant Drosophila line, with a range of different bacteria, the single mutation had effects on both resistance and tolerance, the degrees of which were dependent on the pathogen. Similar findings have been reported by Marsh et al. (2011) in which they showed that the nematode, *Caenorhabditis elegans* which lacked a single lysozyme gene, *LYS-7*, had diminished resistance to *Cryptococcus neoformans*, and other pathogens, but enhanced tolerance to *Salmonella typhimurium*.

Thus, in contrast to the prevailing view that host tolerance genes as opposed to host resistance genes, will inevitably evolve to fixation, it seems more likely that the complexity of host-pathogen interactions will inevitably led to observed variation in both tolerance and resistance traits.

More recently evidence for underlying genetic differences in tolerance and resistance traits has been gleaned from a few experimental animal models—mainly from invertebrates such as Drosophila (Corby-Harris et al., 2007; Ayres and Schneider, 2008, 2009), butterflies (Lefevre et al., 2011), or Daphnia (Graham et al., 2011). However, a study by Raberg et al. (2007) indicated that different strains of mice differed in their tolerance to a *Plasmodium* parasite. Weight loss and anemia were shown to correlate with morbidity or fitness, and the authors used these to identify reaction norms of the slope of host fitness against parasite burden following challenge, in individuals from five genetically distinct strains of mice. They found that, there were significant differences in the reaction norms between mouse strains. This implies that mammals also harbor gene variants that control tolerance traits as well as resistance traits. In addition, they found that the mouse strain that was the most tolerant was the least resistant in terms of peak parasite burden, and *vice versa*, suggesting that reduced tolerance is a cost of resistance—at least in this example. A more recent study has extended these principles to wild fish (Blanchet et al., 2010). However, no genes or mechanisms underpinning these traits have been identified in either of these latter studies. Taken together, all of these studies also suggest that different genes can control disease resistance and tolerance, and that they can be antagonistic. Nonetheless other models suggest that this relationship is not inevitable and resistance and tolerance traits can be independent (Simms and Triplett, 1995; Restif and Koella, 2004). Ayres and Schneider (2008) suggest it is likely that both tolerance and resistance mechanisms need to be evoked to ensure survival, and they propose that a variety of different scenarios could be envisaged for each mechanism ranging from high resistance/low tolerance and *vice versa*, and any state in between.

Thus, genetic variants for resistance and tolerance in populations are likely to depend on previous histories of exposure to pathogens, the types of pathogen and the trade-offs they impose on host fitness. It should therefore be possible to select for resistance, tolerance, or potentially for both traits together in livestock populations, but importantly, the goal will depend on the characteristics of the pathogen and what effect it has on the host. However, the studies described above also indicate that achieving optimum resistance or tolerance to a range of pathogens might prove difficult.

## Genetics of resistance and tolerance in livestock: what needs to be measured?

Identifying the underlying genes for resistance and tolerance in livestock is likely to be difficult, especially in the case of tolerance traits. Nonetheless, evidence that animals also exhibit genetic variation in resistance and tolerance (Raberg et al., 2007, 2009; Blanchet et al., 2010) would suggest that livestock may also harbor selectable gene variants for these traits.

Most studies on genes and their variants related to host responses to pathogens essentially refer to them as disease resistance genes or loci or traits. However, some of these may more correctly be related to tolerance. The problem is that resistance and tolerance are not clearly distinguished and often do not measure appropriate parameters of “fitness” such as growth, weight, health, or reproductive success. Although sheep and cattle breeds and individuals have been described as having variable tolerance to several infections by pathogens e.g., nematodes (Mirkena et al., 2010), and trypanosomes (Noyes et al., 2011), the term tolerance is not well-defined and generally appears to relate to resilience rather than to a demonstration that the tolerant animals' performance and reproductive traits are maintained in the absence of infection, and/or regardless of pathogen burden to a greater degree than non-tolerant animals. This is not to say that, these are not descriptions of tolerance to infection, simply, the relevant parameters have often not been measured. In addition, as argued elsewhere in this special edition (Doeschl-Wilson et al., 2012b), defining tolerance based on groups of individuals makes the estimation of the effect of tolerance traits less accurate, especially for outbred species such as livestock.

In order to distinguish between resistance and tolerance as defense strategies additional data collection is required as pathogen burden also has to be measured, yet it is clear that pathogen burden does not necessarily have a linear relationship with either resistance or tolerance (Viney et al., 2005; Stjernman et al., 2008; Graham et al., 2011). Thus, for example Stjernman et al. (2008) found that, when the fitness cost of host resistance to a parasite is high, then at *both* low and high parasite burden, host fitness costs may be less than at an intermediate parasite burden. Additionally health or performance traits should be measured both pre-infection as well as post-infection as a measure of constitutive health (Graham et al., 2011; Doeschl-Wilson et al., 2012b). This poses further logistical issues as it is not necessarily clear what samples might need to be collected, from which tissues, or at what time following infection. Many pathogens are not easy to detect e.g., *Mycobacteria* spp., and diagnostic tests can be complex with less than optimal specificities and sensitivities (Wadhwa et al., 2012). Nonetheless in order to begin to understand how host genetic variation impacts on resistance and tolerance traits, these relationships between pathogen burden and fitness must be assessed.

In addition to obtaining appropriate data on pathogen burden and its relation to host fitness, in order to ascertain the mechanisms that underlie resistance (or tolerance) in livestock, other parameters of the host response to the pathogen need to be measured. This is not straightforward as often the correlates of protection are not clear, therefore, what the most appropriate parameters to measure can difficult to determine. Immune defenses are highly complex, and the types of protective responses differ between pathogens, making it unlikely that a single parameter will be sufficient to determine the underlying molecular pathways. Furthermore, it is easier to measure some parameters than others e.g., systemic antibody responses can be monitored from small stored serum samples, unlike localized cellular responses in large numbers of animals. Yet for many infections, protective mechanisms involve diverse immune responses that also differ across time (Glass et al., 2012a,b; Leach et al., 2012). Additionally, the relationship between immune measures and protection or indeed, “fitness” is usually not clear (Graham et al., 2010; Schneider, 2011). Cost and logistics generally preclude specific challenge studies using carefully calibrated doses. In large-scale studies, using field data, there are often limited information e.g., only veterinary observations, which reduce the accuracy of any estimates of effect. Furthermore, as pointed out by Graham et al. (2011), simply equating stronger immune responses with greater resistance or tolerance can lead to wrong or even diametrically opposed conclusions. In summary, greater consideration of what to measure is necessary to clearly distinguish resistance from tolerance phenotypes.

## Identity of candidate resistance and tolerance genes

Disease resistance genes are likely to be functional at early stages of pathogen invasion, before it can reach a certain threshold level that would result in host damage. Genetic variants that confer greater resistance should either reduce the pathogen's chances of successful infection or increase the host's rate of pathogen clearance. In contrast, disease tolerance genes should reduce the levels of immunopathology or should enhance the host's ability to protect against pathogen associated toxins. It should be pointed out that even in plants where studies on resistance and tolerance have been undertaken for decades, no specific tolerance genes have been identified to date (Carval and Ferriere, 2010). Although it is now clear that variation in relative resistance and tolerance traits in individuals and strains exist in animal species, for the main part polymorphisms that underpin these traits have not been identified. However, it would seem likely that components of host defense against micro-organisms are likely to play a role. In the next sections a consideration of what molecules and pathways the host employs to defend itself against infection and how they may underpin resistance and tolerance traits. Potential pathogens must first cross the interfaces between the host and its environment. These interfaces consist of the skin and cells lining the mucosal surfaces of the gut, respiratory tract, mammary gland, and genital tract.

### Host receptors

In order to gain access to the host environment, a pathogen generally does so by binding to host cell surface molecules. One example, where variation in the genes encoding receptors results in host resistance is the C-C chemokine receptor type 5 (CCR5) gene, which is associated with resistance to human immunodeficiency virus (HIV) (Reynes et al., 2001). CCR5 is a chemokine receptor expressed on certain immune cells, but also acts as a receptor for the HIV virus to enter cells. Individuals with a deletion mutation express lower levels of the receptor, which results in lower levels of viral entry, and also in less risk of progression (Reynes et al., 2001). Generally, these individuals are healthy suggesting that this receptor is not essential, but some studies have suggested that lack of it may have a detrimental effect on responses to other pathogens (Dean et al., 2002). Unfortunately, CCR5 is not the only receptor for HIV entry. However, as this mutation reduces the initial infectious dose of HIV, thus lowering the risk of infection and progression, it can be considered as a canonical “resistance” gene.

Many pathogens gain entry via host receptors, but in the case of livestock the majority are unknown. An exception is the gut receptor for *E. coli* F18 encoded by the *fut1* gene in pigs, which confers complete resistance to *E. coli* (Meijerink et al., 1997). In cattle it is known that foot and mouth disease virus (FMDV) enters cells by attaching to the bovine cell surface integrin, αυβ6 (Monaghan et al., 2005), but whether there are variants that confer resistance is not known. The bovine viral diarrhea virus (BVDV) employs the bovine CD46 cell surface molecule, a member of the complement regulatory receptors, to gain entry to cells (Maurer et al., 2004). Recently, genetic variants in bovine CD46 have been shown to influence cell permissiveness for BVDV, at least *in vitro* (Zezafoun et al., 2011), and thus carriers of CD46 alleles might vary in resistance to BVDV.

### Host detection of pathogens and danger

Once a pathogen has breached the initial barriers to its entry, the host has a small window of opportunity to detect its presence before it begins to replicate. Therefore, it is of no surprise that the skin and mucosal tracts contain both non-immune cells such as fibroblasts, epithelial and endothelial cells, as well as many innate immune cells which all act as sensors of pathogens and which are highly effective at signaling alarm to the rest of the immune system (Zarember and Godowski, 2002; Matzinger, 2007). Innate defense mechanisms exist in all metazoan species and represent ancient evolutionary protection strategies that probably, first developed in the last common ancestor between animals and plants, even though plants do not contain specialized immune cells (Ronald and Beutler, 2010). These detection systems function to discriminate self from non-self [as originally proposed by Janeway (1989) whose farsighted view was to hypothesize the existence of pattern recognition receptors (PRRs)] and/or to discriminate between agents of potential damage from those which are benign [the “danger” theory as proposed by Matzinger (1994)]. The consensus view, currently, is that both pathogen and non-pathogen associated damage or danger signals may in fact be necessary for initiation of responses to pathogens and that the context in which pathogens are detected is critical for the ensuing innate immune response (Fontana and Vance, 2011). Since both types of signal are crucial determinants of the strength and nature of the ensuing response to inflammatory signals, they need to be considered as elements of both resistance and tolerance.

Among the earliest detection, systems are pattern or pathogen recognition receptors (PRRs) and molecules (PRMs), which are expressed in most cell types, but importantly in the cells of mucosal surfaces such as epithelial cells and fibroblasts, as well as immune cells such as the antigen presenting cells (APCs), macrophages, and dendritic cells. These receptors recognize conserved molecular structures on pathogens that are not found in hosts, pathogen associated molecular patterns (PAMPs), for example, bacterial cell wall components and double stranded RNA (Kumar et al., 2011). PRRs are present as soluble molecules as well as on cell surfaces and within the cytoplasm and thus can detect both extra- and intra-cellular pathogens (Kersse et al., 2011). An example of an early detection system involving soluble proteins are the trypanolytic factors present only in human and some primate serum, which renders them resistant to most trypanosome species (Vanhollebeke and Pays, 2010). These factors involve apolipoprotein L-1 (APOL-1), which is related to the family of pro and anti-apoptotic Bcl2 molecules and evolved in early primates from a gene duplication event, and an acute phase protein, the soluble PRR, haptoglobin-related protein (Hpr). Sera from other host species such as Cape buffalo also contain factors which kill trypanosomes, but the so-called trypanotolerance trait present in certain breeds of African cattle involves other components of the innate immune system (Namangala, 2012).

PRRs consist of various domains with a variety of functions including ligand recognition, which are highly evolutionarily conserved (Ronald and Beutler, 2010; Hansen et al., 2011). The best known examples of PRR are the family of toll-like receptors (TLR), and subtle differences in sequence across species and within species have been associated with differences in response to a variety of pathogens in many species including livestock (Werling et al., 2009). Indeed we have suggested that they may be the best candidates for selection of animals with lower risk of infections (Jann et al., 2009). In addition, we (and others) have identified signals of positive selection in bovine TLRs (Jann et al., 2008; Smith et al., 2012). However, many other PRRs exist including the C-type lectin receptors, as well as sets of receptors which are important for detecting the presence of cytosolic PAMPs such as RNA and DNA including NOD-like receptors (NLRs), RIG-I-like receptors (RLRs), and DNA receptors (cytosolic sensors for DNA). There are likely more to be discovered and the degree to which they all interact (after all pathogens have many different PAMPs) is still a hot topic of research (Kawai and Akira, 2011).

It also appears that the host is primed to respond to intrinsic inflammatory signals, sometimes referred to as danger or damage signals (Matzinger, 2002; Bianchi, 2007; Lotze et al., 2007). These are essentially host components released or expressed immediately following stress and damage of cells and tissues, and which evoke an inflammatory response. The nature of these intrinsic alarm molecules, sometimes referred to as “alarmins” and their receptors is still not well-understood and their role in pathogen or trauma-induced inflammation remains controversial (Manson et al., 2012).

The term damage-associated molecular patterns (DAMPs) thus can encompass both PAMPs and alarmins (Bianchi, 2007). The receptor candidates for alarmins include PRRs themselves (Seong and Matzinger, 2004), the Receptor for Advanced Glycation End products, RAGE, (Bianchi, 2007), and an APC receptor, CLEC9, recently discovered to recognize actin filaments released by necrotic cells (Ahrens et al., 2012).

### Host innate responses

Such a two signal model would help explain, why hosts respond to pathogenic micro-organisms but not commensals, which also express PAMPs (Nussbaum and Locksley, 2012). In a recent review, Blander and Sander (2012) suggest that recognition and response are likely to involve a whole series of interacting components, and the type and magnitude of response depends on at least five checkpoints. These enable the host to assess the threat of the micro-organisms to host integrity and respond accordingly. First, as micro-organisms have multiple PAMPs, the host initially integrates these signals, resulting in a cascade of intracellular signaling through various adapter molecules (e.g., MyD88), followed by activation of the MAP kinase family, which in turn switches on transcription factors such as NF-kB and interferon regulatory factor (IRF) family members. These down-stream signaling pathways, which are conserved across species, result in the up-regulation of molecules associated with inflammation such as cytokines, and/or induction of autophagy and various cell death pathways leading to synergistic production of cytokines (Bianchi, 2007; Duprez et al., 2009; Hansen et al., 2011). If the host's innate responses are strong and rapid, there is evidence that micro-organisms are cleared and little pathology will be evident (Evans et al., 2010). Any cell death will be well controlled through apoptotic mechanisms, which ensure that the cellular contents are not released to the external milieu. The resulting apoptotic bodies are then engulfed by phagocytic cells such as macrophages (Mφ) (Duprez et al., 2009). Thus, genes controlling these first few hours of response to pathogen invasion are likely gene candidates for resistance traits. However, if this does not result in elimination or destruction of the micro-organism, then the tissue load (Willer et al., 2012), and whether the micro-organism is alive or dead (Fontana and Vance, 2011), in other words sensing micro-organism growth in tissues, may become more important determinants of immunopathology. In this second phase, the host may detect micro-organism derived metabolic molecules such as mRNA or bacterial pyrophosphates. The third checkpoint may be the sensing of virulence factors or their activity, although it has to be said that commensals can also possess virulence factors such as type III secretion systems, and what may count more is whether micro-organisms have breached the mucosal layer (Swiatczak et al., 2011). Possibly hosts actually sense changes in their own metabolism as well, for example changes in transcription and translation (Kleino and Silverman, 2012). One key component appears to be the up-regulation of a transcription factor, ZIP-2, and transcription of its target genes, including infection response gene-1 (irg-1), at least in the nematode, *C. elegans*. Other key host perturbations may include pathogen driven rearrangements of the cytoskeleton and it is possible that some of the cytosolic PRRs such as the NLRs and DNA sensors may be involved in recognition of these metabolic changes (Vance et al., 2009). Triggering of these cytosolic PRRs adds a further ratcheting up of responses by inducing inflammasome formation and the activation of caspases. These enzymes in turn activate and release the alarmin, interleukin 1β. Inflammatory cell death (necrotic or pyroptotic) (Duprez et al., 2009) leads to the release of other alarmins and up-regulation of type I interferon pathways (Kersse et al., 2011). Thus, as the second and third checkpoints are breached, the level of the inflammatory response increases significantly. Since many of these host processes are part of cellular or tissue homeostasis, and also potentially highly immunopathogenic, one might argue that gene variants encoding the major regulators during these two phases would be prime candidates for “tolerance” genes. However, their identities are only just becoming clearer and many remain controversial.

A further checkpoint is the detection of invasion from compartments that allow colonization, for example the lumen of the gut, into sterile tissues. Such invasion could occur through tissue injury or expression of virulence factors. This transfer into a new host niche exposes invading micro-organisms to the attention of innate immune cells especially, phagocytes including neutrophils, and the myeloid APC, Mφ, and dendritic cells (DC). These are primed for scaling up effector mechanisms including phagocytosis, cell death and release of defense-related molecules. Mφ are perhaps, pivotal cells during this phase as they have an extensive range of effector functions which depending on their microenvironment, include phagocytosis, scavenging, cytotoxicity, and production of pro- and anti-inflammatory cytokines (Gordon and Taylor, 2005; Plueddemann et al., 2011).

In mice and humans various APC subsets have been identified including M1 and M2 Mφ, which are involved in inflammatory signals and tissue repair respectively, but these classifications are less clear in livestock species (Gordon and Taylor, 2005; Fairbairn et al., 2011; Murray and Wynn, 2011). In particular, Mφ and other phagocytes discriminate between host cells that have undergone different types of cell death and produce a range of immunomodulatory molecules that determine, whether inflammation develops or tissue repair occurs (Poon et al., 2010). Thus, Mφ phagocytosis of cells in the early stages of apoptosis tends to result in the production of factors such as vascular endothelial growth factor (VEGF) and transforming growth factor-β (TGF β), which result in tissue repair and down-regulation of inflammation i.e., Mφ function to return and maintain tissue homeostasis. Phagocytosis of cells undergoing apoptosis by DC results in tolerance (as per its immunological definition) to self. In contrast, phagocytosis of cells in the late stages of apoptosis or those that have undergone inflammatory forms of cell death (necrosis or pyroptosis), result in Mφ producing pro-inflammatory cytokines and further immunopathology. However, this is an over-simplification, and Mφ (and DC) express a wide-range of intermediate phenotypes, apart from those associated with M1 and M2 Mφ in response to internal and external purturbations, whether from micro-organisms or intrinsically derived signals.

Thus, the degree of inflammation and other responses is determined by the different temporal and spatial interactions between host and micro-organism. These processes in turn result in recruitment of further immune cells to the site of infection and ultimately induction of the adaptive response, at least in jawed vertebrates.

### Initiation of host adaptive responses by the innate immune system

Most of the studies that have identified that genetic variation in resistance and tolerance exists in animals have been conducted in species that do not have adaptive immune systems (Graham et al., 2011; Ayres and Schneider, 2012). One might argue that disease resistance or tolerance genes would not be functional during the adaptive immune response, because it takes several days for an adaptive immune response to develop as it is dependent on the innate immune response, which both drives and directs the adaptive response. Without an innate response, no adaptive immune response occurs. In addition, the cells and molecules of the adaptive response also influence the innate immune response. Thus, it seems likely that resistance and tolerance traits may also be expressed as part of the link between the innate and acquired immune system. Indeed, the best known of all the immune-related candidates for disease resistance are the highly polymorphic major histocompatibility complex (MHC) classical genes, MHC I and MHC II (Hill, 2012), which are crucial for presentation of pathogen derived antigen to T cells, without which no induction of pathogen specific adaptive immunity would occur. Infectious disease associations with these loci abound in livestock studies (e.g., see reviews on cattle, pigs, and chickens respectively, Lewin et al., 1999; Lunney et al., 2009; Calenge et al., 2010).

The key players in linking the innate to the adaptive immune system are APC, Mφ, and DC (Hume, 2008; Segura and Villadangos, 2009), which directly initiate the adaptive immune response through interactions with T cells. Depending on the microenvironment and pathogen, APC, in conjunction with MHC class I and class II presentation of pathogen derived peptides to T cells, also express different cytokines and other cell surface molecules which provide the context or second signals that result in the activation of functionally distinct subsets of T cells. Thus, initiation of an adaptive response also relies on the early interactions with pathogens and other intrinsic “danger” signals to provide the second signal along with antigen presentation. As with APC, these T cell subsets have well-studied phenotypes in mouse and human with specific roles in inflammation and host immunity, regulation, and suppression of uncontrolled inflammation (Nakayamada et al., 2012), but are less well-described for livestock.

In brief, intra-cellular infectious agents together with accompanying inflammation invoke the classical inflammatory M1 Mφ to produce IL12 and IL23, which provide the second signals that prime Th1 and Th17 cells respectively. These cells which are involved in amplifying the functions of and induction of cytotoxic T cells through the actions of their signature cytokines, interferon-γ (IFNγ) and interleukin (IL)-17 respectively. However, Th17 cells which reside in the epithelial layers are not only pro-inflammatory, they also help to restore barrier function following inflammation through their production of the tissue protectant IL-22 (Ouyang et al., 2011; Akdis et al., 2012). Since M1 cells are also major producers of pro-inflammatory cytokines and ROS, they also have the potential to cause tissue damage.

In contrast, extracellular and metazoan pathogens invoke M2-induced Th2 cells, which produce B cell help factors such as IL4 and IL13. In addition, Th2 cells (together with M2 Mφ) also have a regulatory role in limiting and resolving Th1 type inflammation, a role in tissue repair and potentially a role in tolerating the presence of metazoan micro-organisms (Martinez et al., 2009; Allen and Wynn, 2011). Although mouse strains can differ in their propensity to develop M1 vs. M2 response phenotypes (Mills et al., 2000), there has been very little investigation into the identification of the underlying gene variants. One recent paper has suggested that polymorphisms in the transcription factor, interferon regulatory factor 5, which lead to autoimmunity, may be linked to its role in promoting the induction of M1 cells (Krausgruber et al., 2011). Although it might be tempting to suggest that genetic variants which predispose animals to make an M2/Th2 associated response might enhance their tolerant phenotypes, in fact these cell types are also inflammatory (Jenkins et al., 2011). Furthermore, mouse strains with a Th2 type propensity to respond to pathogens are more susceptible to pathogens that require a Th1 type response for adequate protection (Mills et al., 2000). Nevertheless, further understanding of genetics which may underlie a predisposition to macrophage polarization into distinct phenotypes may be very instructive for influencing the genetics of resistance and tolerance to prevailing micro-organisms in livestock species.

### Host adaptive immune responses

Apart from interactions between the innate and the adaptive immune systems, it is also possible that resistance and tolerance genes may be associated with the main players in the acquired immune response, namely the T and B cells. Although it has been assumed that somatic recombination of antibody and T cell receptor (TCR) genes generates very high and similar levels of diversity of pathogen recognition in all individuals, genetic differences between individuals remains a possibility. Firstly, the inherent, constitutive or basal level of immunity will also encompass the components of the adaptive immune system (Clapperton et al., 2009) and therefore this must have a separate associated cost to the host organisms, further complicating the picture with respect to life history trade-offs. Secondly, it was argued that the high potential diversity of antibody and TCRs should mean that all individuals in a species would not be limited in terms of recognition of foreign antigen because of “holes in the repertoire,” i.e., be unable to respond because of deficiencies in the germline components of antibody or TCR genes, or because of elimination of self-reactive T or B cells (Goodnow, 1996). However, viral escape through mutation of viral T cell epitopes has been described, whereby selection in the host appears to favor the appearance of viral mutations that presumably mimic a self-peptide, thus abrogating T cell responses and leading to chronic persistence of the virus (Wolfl et al., 2008). In contrast, a common deletion in the TCR beta-chain locus in humans actually leads to enhanced responses to a virus (Brennan et al., 2012). Thus, it is possible that differences in the TCR repertoire could account for some of the variation in responsiveness in livestock species.

Several other distinct T cell subsets have been described including various regulatory T cell subsets (Tregs) and these provide a further interacting level of immune response that influences the final outcome of pathogen or other insult (Vignali et al., 2008). Their signature cytokines include IL-10 and TGFβ, which are anti-inflammatory and thus they are important in regulating inflammation, and tolerance.

Clearly the components of the acquired immune system cannot be ignored in terms of the genetics of resistance and tolerance in higher vertebrates. However, although adaptive immunity is specific to the pathogen, the initiating responses are much less discriminatory and are more directed by the type and spatial location of the pathogen.

### Anti-inflammatory processes, resolution, and tissue repair

If this cascade of events results in elimination of the invading micro-organism, then resolution of inflammation will occur, partly because of removal of the stimuli, but also because a set of negative feedback loops are also set in motion that control the extent of inflammation. If elimination of pathogens does not occur the result will be chronic inflammation or even mortality because of an over-whelming cytokine storm (Tisoncik et al., 2012). However, the field of anti-inflammation, repair, and resolution is a growing area of research and many new factors and pathways remain to be discovered.

Resolution includes immunoregulatory and tissue repair pathways, but these are interdependent with the pathways leading to inflammation (Serhan and Savill, 2005; Shields et al., 2011) and their activation may result in a return to homeostasis or more permanent tissue damage such as scarring and fibrosis. An important point is that, many of these resolution and regulatory pathways are triggered by the same receptor generated signals as inflammation itself. In fact, intact PRR signaling has been shown to be essential for maintenance of tissue homeostasis and tissue repair (Lawrence et al., 2001; Rakoff-Nahoum et al., 2004; Jiang et al., 2005). In a mouse model of *Citrobacter rodentium* infection in the gut, Bergstrom et al. (2012) suggest that resistance and tolerance are interlinked through TLR and NLR-based mechanisms. In a *Drosophila* model, a homolog of the MAP kinase family, p38, which conventionally is regarded as part of the signaling process for defense, has been linked to tolerance through a role in phagocytosis (Shinzawa et al., 2009).

Shields et al. (2011) have also proposed that resolution may involve the recognition of resolution-associated molecular patterns (RAMPs). They suggest that these are endogenous molecules expressed and released when cells are necrotic or stressed and include heat shock proteins which are involved in the cellular translocation of proteins, as chaperones in the correct folding of newly synthesized protein in the endoplasmic reticulum and also in targeting proteins for the proteasome for degradation.

Specific down-regulation of inflammation involves the induction of anti-inflammatory molecules such as IL-10, TGFβ, IL-1R antagonist, suppressors of cytokine signalling (SOCS), peroxisome proliferator-activated receptor (PPAR) ligands, tyrosine phosphatases SH2-containing phosphatase 1 (SHP-1) and many others, at least partly through the induction of Tregs (Straus and Glass, 2007; Yoshimura et al., 2007; Ouyang et al., 2011; Shields et al., 2011). Recent research has suggested that induction of an IL-6 cytokine family member, leukemia inhibitory factor (LIF) leads to STAT3 signaling and may control tissue repair in lungs of mice suffering from pneumonia (Quinton et al., 2012). LIF has previously been associated with stem cell maintenance and this function may be operating in this situation by promoting cell proliferation or limiting cell death, suggesting further avenues for identifying new tolerance genes.

However, resolution of inflammation is not simply an anti-inflammatory suppressive process, but involves an active process in which specific metabolites are biosynthesized from essential fatty acids in epithelial cells and macrophages in response to inflammation. They include lipoxins, resolution-phase interaction products (resolvins), protectins, sphingosine-1-phosphate, and Maresin 1 (Rivera et al., 2008; Serhan et al., 2008, 2012). These mediators induce uptake and clearance of dead cells and pathogens and they stimulate tissue regeneration and are thus important for homeostatic mechanisms. They are synthesized through cyclooxygenase, lipoxygenase, and epoxygenase pathways and many of the intermediates and products of these pathways are anti-inflammatory and act through PPAR transcription factors (Wahli and Michalik, 2012). PPARs signaling down-regulates the inflammatory transcription profile by suppressing the activity of inflammation-responsive transcription factors including NF-kB. They also maintain an M2 Mφ phenotype in tissues, promoting tissue maintenance activities through M2 Mφ role in scavenging, angiogenesis, tissue remodeling, and repair (Martinez et al., 2009). Given the role of PPARs, M1 and M2 cells in adipocyte, fatty acid and energy metabolism (Shapiro et al., 2011), their modulation could have very important bearing on resistance and tolerance traits, but again much remains to be discovered about them.

Thus, it should be emphasized that the mechanisms controlling anti-inflammatory processes, resolution, and tissue repair play an essential role in regulating the host response to invading pathogens at all levels from the initial recognition of pathogen presence to the triggering of the innate and acquired immune mechanisms. The genes involved cannot be easily categorized as simply part of tolerance traits but are intimately linked to resistance traits as well.

## Identification of loci and genetic variants for resistance and tolerance

So what is the evidence that different genetic variants exist for resistance and tolerance in complex metazoans? Some recent evidence is pertinent and will illustrate the difficulties of consigning genes to one or the other trait.

As previously discussed there is evidence that, there is a genetic component that underlies variation in resistance and tolerance. Although it is difficult to demonstrate directly that hosts and pathogens co-evolve, the general consensus is that evidence for this can be seen in changes in frequencies of genes in populations exposed to specific pathogens (Allison, 1954; De Campos-Lima et al., 1994; Novembre and Han, 2012) and in terms of signals of positive selection in genomes (Fumagalli et al., 2011) with innate immune genes having the strongest signals (Barreiro and Quintana-Murci, 2010). It has to be said that these types of analyses are more difficult when attempting to include non-model species with relatively poor annotation such as livestock (Brieuc and Naish, 2011) with limited data on frequencies of candidate genes in different populations. The author and others have shown that positive selection in immune related genes can be detected, but obtaining evidence that positively selected sites are important functionally in livestock is more difficult (e.g., Jann et al., 2008; Smith et al., 2012). In addition, most if not all studies on candidates concentrate on defense, and not on tolerance. Thus, it remains to be seen if similar differences in frequencies or evidence of positive selection can be found for livestock or indeed any other population.

The most convincing example of pathogen driven selection of host genes, is the interaction of human populations with *Plasmodium falciparum* where frequencies of gene variants associated with malaria resistance and tolerance are at significantly higher levels in areas where malaria is prevalent than in non-endemic parts of the world (Durand and Coetzer, 2008). Thus, in the case of malaria, homozygosity for the hemoglobin mutation that causes sickle cell anemia (HbS), is associated with loss of fitness or reproductive success, which acts as a counter balance, preventing the sickle cell gene from going to fixation in a population where malaria is endemic (Allison, 1954). Recently, it has become apparent that the HbS gene confers both resistance and tolerance to the malaria parasite (Ferreira et al., 2011). In addition, Seixas et al. (2009) have identified a mechanism that confers tolerance to malaria, which involves the induction of heme oxygenase-1 (HO-1) in the liver of a tolerant, but not in a susceptible mouse strain. HO-1 is a tissue protectant which degrades free heme released from hemoglobin in *Plasmodium* infected red cells. Free heme leads to the production of ROS, resulting in apoptosis of liver cells. The underlying genetics that leads to differential HO-1 expression in more or less tolerant mouse strains remains unclear. However, in humans, polymorphism in the promoter region of HO-1 controls its expression and is associated with severe malaria (Walther et al., 2012). Again, whilst it might seem attractive to target HO-1 as a potential candidate gene for tolerance, this may be counterproductive as induction of HO-1 during malaria infection resulted in a fatal reduction in resistance to *Salmonella* in mice (Cunnington et al., 2012).

In a unique study, Miyairi et al. (2012) have specifically investigated the genetics of resistance and tolerance to *Chlamydia psittaci* in a range of genotyped mice. The authors measured weight loss and pathogen burden following infection and identified a genetic locus on mouse chromosome 11 (*Ctrq3*), which influenced pathogen load i.e., resistance as well as weight loss and thus suggest that the same locus controls resistance and tolerance. They have circumstantial evidence that likely candidate genes belong to the family of immunity-related GTPases (IRG). These genes encode proteins which are highly up-regulated in pathogen containing cells such as macrophages (Taylor et al., 2007). They localize to pathogen containing vacuoles and are involved in processing of pathogens through destructive pathways such as autophagy resulting in the elimination of the pathogen. Bearing in mind that the authors measured a limited number of traits, they presented evidence that the *Ctrq3* locus also controls macrophage activation and neutrophil accumulation at the site of infection, but these traits also independently influenced weight loss. Macrophage activation also had an independent effect on pathogen burden. These intriguing results again point to the importance of macrophages in determining tolerance and resistance traits.

As discussed previously in livestock species even where appropriate parameters have been measured, their relationships have not been explored. An exception is a paper by Zanella et al. (2011) who have defined loci for tolerance to infection with *Mycobacterium avium* subspecies *paratuberculosis* (MAP), by measuring MAP fecal shedding as a measure of fitness and MAP levels in tissues as a measure of infection intensity. It may be instructive to revisit previously published data to explore the components of resistance and tolerance in livestock responses to pathogens. For example, in the author's own research a *Bos indicus* cattle breed, Sahiwal, has been shown to be more resistant to a tropical tick-borne protozoan parasite, *Theileria annulata* than a non-tropical breed, the Holstein (Glass et al., 2005). Both breeds became infected following experimental challenge, but only the Sahiwals survived to the end of the experiment, the Holsteins being overcome with an overwhelming inflammatory response. The parasite infects Mφ which also plays an important role in the inflammatory response as well as protection against the parasite. Among other transcriptional differences between the two breeds (Jensen et al., 2008), two molecules stand out: signal regulatory protein beta (SIRPβ), and TGFβ2 (Chaussepied et al., 2010; Glass et al., 2012b). Both are involved in regulation of inflammation and both were more highly expressed in Holstein Mφ than Sahiwal Mφ. TGFβ2 appears to be associated with greater virulence and also higher propensity for invasion (Chaussepied et al., 2010). Given these intriguing differences, reconsideration of the original data in terms of regressing health against pathogen levels in the two breeds, is warranted as the pathogen burden was less in the Sahiwals, and some parameters of fitness (temperature and packed cell volume) and clinical, hematological, and inflammatory related responses were measured before and during the experimental trial.

## Conclusions

In summary, although genetic resistance and tolerance are likely underpinned by distinct mechanisms, their initiation is likely to be intertwined and the outcome of host-pathogen interactions is dependent on both the host and pathogen characteristics. Pathogens have evolved very distinct strategies to ensure their reproductive success, which is dependent on their ability to thrive in their host species and to transmit to other individuals. Some pathogens produce factors which cause toxicity in their host, or induce a dysregulated inflammatory response which can prove fatal. Host metazoans that can overcome such pathogens, and survive may adopt two distinct strategies, first: quickly eliminate such pathogens; second, tolerate the effects by producing anti-toxins for example, or employ stronger or faster acting negative feedback loops to prevent inflammation damaging the tissues. Although these strategies appear to be distinct, in fact the complex host processes encompassing these tactics are intimately entwined. Thus, distinct host resistance and tolerance traits may be less common than traits that involve elements of both strategies which are likely to have evolved together to overcome infectious threats. Pathogens can also manipulate the host response to their own gain, for example driving recruitment of immune cells that provide cellular niches for the dissemination of the pathogens. Pathogens have also evolved strategies to overcome, evade and subvert the host defense mechanisms, and instead of their removal, infection may become chronic; such a scenario may result in reduction in fitness of the host, but is not inevitable. Much remains to be discovered especially the genes and pathways controlling anti-inflammatory responses, resolution and tissue repair. A better understanding of these mechanisms and their relationship to inflammation and the pathogen driven host defense responses, especially in livestock species, is clearly needed before we can begin to breed livestock for increased resistance and/or tolerance.

### Conflict of interest statement

The author declares that the research was conducted in the absence of any commercial or financial relationships that could be construed as a potential conflict of interest.
